# Regulatory T cells in vitiligo: a review of functional disequilibrium between peripheral blood and lesional tissue

**DOI:** 10.3389/fimmu.2026.1844106

**Published:** 2026-05-18

**Authors:** Ye Qiu, Yurong He, Fang Liu

**Affiliations:** Department of Dermatology, Beijing Chaoyang Hospital, Capital Medical University, Beijing, China

**Keywords:** biomarker, immune tolerance, JAK inhibitors, lesional skin, peripheral blood, regulatory T cells (tregs), treg−melanocyte interaction, vitiligo

## Abstract

**Introduction:**

Vitiligo is an autoimmune skin disorder driven by CD8^+^ T cell−mediated melanocyte destruction, in which breakdown of immune tolerance plays a pivotal role. Regulatory T cells (Tregs) are central to maintaining peripheral immune tolerance, but their abnormalities in vitiligo remain insufficiently integrated from a systemic perspective.

**Methods:**

This review synthesizes published evidence on Treg biology and immunoregulatory functions, focusing on comparative analyses between peripheral blood and lesional skin of vitiligo patients. We also examine Treg−melanocyte interaction networks, Treg−targeted therapies, and the potential of Treg−related biomarkers.

**Results:**

In peripheral blood, vitiligo patients exhibit reduced Treg frequency, impaired suppressive capacity, and Th1-like polarization, alongside dysfunctional subsets such as STAM (signal-transducing adaptor molecule)^+^ Treg and exhausted memory Treg. In lesional skin, tissue−resident and antigen−specific Treg subsets are markedly decreased, and the proinflammatory microenvironment further compromises their function. This systemic−to−local Treg dysregulation leads to insufficient suppression of autoreactive CD8^+^ T cells and persistent melanocyte destruction. Mechanistically, breakdown of the protective Treg−melanocyte network involves systemic Treg deficits, failure of local tolerance, melanocyte−intrinsic death programs, and disrupted homing/signaling. Therapeutically, NB−UVB, JAK inhibitors, and low−dose IL−2 show promise in restoring immune balance, and emerging Treg subsets may serve as biomarkers for treatment response.

**Discussion:**

The anatomical hierarchy of Treg dysregulation—from blood to skin—underscores that effective immunosuppression requires not only sufficient Treg numbers but also precise tissue localization and functional adaptation. Future single−cell transcriptomics and functionally defined Treg subsets may facilitate translation of Treg−based biomarkers and therapies into practice.

## 1 Introduction

Vitiligo is an acquired autoimmune skin disorder characterized by localized or generalized cutaneous depigmentation, with a global prevalence of approximately 0.5%–2% (1), and it profoundly affects patients’ physical appearance and quality of life. Its central pathological mechanism involves cytotoxic CD8^+^ T cell-mediated autoimmune attack against melanocytes. Increasing evidence in recent years has indicated that dysregulation of the immune regulatory network—particularly the imbalance between excessive activation of effector T cells (Teff) and insufficient function of regulatory T cells (Treg)—is a critical driver of disease initiation and persistence. As a key cellular subset responsible for maintaining peripheral immune tolerance, abnormalities in the number, function, and tissue distribution of Treg have been widely documented in patients with vitiligo. This review systematically summarizes the biological characteristics and functions of Treg, comprehensively examines their aberrant manifestations in the peripheral blood and lesional skin of vitiligo patients, analyzes the mechanisms underlying the breakdown of the protective interactive network between Treg and melanocytes, and further discusses Treg-targeted therapeutic strategies and biomarker research perspectives, with the aim of providing a theoretical basis for a deeper understanding of vitiligo immunopathology and for the development of novel immune-based interventions.

## 2 Biological characteristics and functions of treg

### 2.1 Biological characteristics of treg

Regulatory T cells (Treg) constitute a specialized lineage of CD4^+^ T lymphocytes, characterized primarily by the stable expression of the lineage-defining transcription factor FOXP3, high expression of the high-affinity interleukin-2 (IL-2) receptor CD25, upregulation of inhibitory molecules such as cytotoxic T-lymphocyte-associated protein 4 (CTLA-4), and concomitantly low expression of CD127. This coordinated transcriptional and phenotypic program collectively determines the lineage stability and immunosuppressive potential of Treg. Treg are mainly derived through two developmental pathways: one is thymus-derived Treg (tTreg), which establish immune tolerance through selective recognition of self-antigens; the other is peripherally induced Treg (pTreg), which differentiate from conventional CD4^+^ T cells under teloregion conditions, particularly in the presence of transforming growth factor-β (TGF-β) (2). In adults, Treg exhibit marked functional heterogeneity and can be distributed along a continuum from “resting” to “activated” states, with effector Treg being transcriptionally, metabolically, and epigenetically adapted to inflammatory tissue environments. Notably, tissue-resident Treg additionally acquire tissue-specific programs of chemotaxis, retention, and survival, suggesting that the biological characteristics of Treg are determined not only by lineage commitment but also profoundly shaped by the tissue microenvironment (3). Traditional investigations of Treg heterogeneity have largely relied on flow cytometry, whereas the recent application of single-cell RNA sequencing (scRNA-seq) has greatly deepened our understanding of Treg subset diversity. Using scRNA-seq, Xiao et al. identified a STAM^+^ Treg subset in the peripheral blood of patients with progressive non-segmental vitiligo. This subset highly expressed genes such as *RTKN2*, *IKZF2*, and *IL2RA*, and STAM, as a signal-transducing adaptor molecule, may influence Treg function through regulation of the JAK–STAT signaling pathway. This finding reveals a novel layer of transcriptional heterogeneity of Treg in vitiligo (4).

### 2.2 Immunoregulatory functions of treg

Treg exert immunosuppressive effects through multiple complementary and non-redundant mechanisms, thereby effectively restraining autoreactive immune responses (5). Among these, CTLA-4-mediated regulation of antigen-presenting cells represents one of the key pathways, as it reduces the co-stimulatory capacity of antigen-presenting cells and suppresses the activation of effector T cells (Teff) (6). Treg can also secrete immunosuppressive cytokines, such as transforming growth factor-β (TGF-β) and interleukin-10 (IL-10), thereby attenuating inflammatory signaling and promoting tissue tolerance. In addition, the constitutively high expression of CD25 enables Treg to efficiently consume IL-2, thus limiting the expansion and survival of Teff through a mechanism of “growth factor deprivation” (7). Beyond soluble mediators, Treg also exert local immunosuppressive effects within tissues through cell contact-dependent interactions and metabolic regulation, underscoring the spatial dependence of immune regulation (8). Therefore, effective immunosuppression depends not only on the number of Treg but also on their precise localization within target tissues and the stability of their suppressive function (9). In inflammatory skin diseases such as vitiligo, failure to establish a local Treg regulatory niche—owing to impaired recruitment, retention, or insufficient adaptation to an IFN-γ-dominated inflammatory microenvironment—may permit persistent activation of cytotoxic T cells even in the presence of circulating Treg, thereby resulting in functional disequilibrium between the peripheral blood and lesional tissue (10).

## 3 The critical role of treg in the immunopathogenesis of vitiligo

The immunopathology of vitiligo is centered on cytotoxic CD8^+^ T cell-mediated anti-melanocyte responses. A large body of evidence has demonstrated that IFN-γ-driven inflammatory signaling occupies a central position in both disease initiation and maintenance: activated CD8^+^ T cells secrete IFN-γ, which, through the JAK/STAT signaling axis, induces keratinocytes and fibroblasts to express CXCL9 and CXCL10, thereby continuously recruiting CXCR3^+^ effector T cells (Teff) into the skin and establishing a self-amplifying inflammatory loop (11). This “IFN-γ–chemokine–Teff” axis is regarded as a key histological feature of the immune microenvironment in vitiligo lesions and also constitutes an important mechanistic basis for sustained melanocyte injury.

Concomitant with persistent activation of effector immunity, insufficient Treg-mediated negative immune regulation represents another central dimension of immune disequilibrium in vitiligo. As a key cellular population responsible for maintaining peripheral immune tolerance and restraining autoreactive immune responses, abnormalities in the number and/or function of Treg have been repeatedly reported in patients with vitiligo. The number and functional status of Treg in peripheral blood and skin tissue were systematically evaluated in a meta-analysis that integrated multiple studies comparing patients with vitiligo and healthy controls (12). Their findings demonstrated that patients with vitiligo exhibited reduced Treg levels and impaired suppressive function across different anatomical sites, with this dysregulation being particularly pronounced in lesional skin, suggesting that disruption of Treg-mediated immune tolerance constitutes an important component of vitiligo immunopathogenesis. More importantly, recent studies have increasingly recognized that the immunosuppressive effects of Treg are not determined solely by their abundance in peripheral blood, but are highly dependent on their recruitment, precise localization, and functional maintenance within the skin. Using a murine model of vitiligo, a study found that Treg cells directly contacted Teff cells at lesional sites, and that the degree of Treg recruitment to the skin was negatively correlated with disease severity (13). However, Treg abundance in peripheral lymph nodes did not predict this regulatory effect, thereby underscoring the importance of local Treg localization. When the IFN-γ–CXCL9/CXCL10 axis is markedly enhanced while Treg-mediated suppressive feedback is insufficient, local immune responses are more likely to shift from a controllable inflammatory state to a self-sustaining chronic autoimmune attack.

Therefore, Treg–Teff imbalance may be regarded as the structural core of immune dysregulation in vitiligo: pathogenic Teff cells, particularly CD8^+^ T cells, become excessively activated and are continuously recruited to the skin, while the local Treg-mediated regulatory network fails to be effectively established, ultimately leading to persistent melanocyte destruction (10).

## 4 Studies on peripheral blood treg in vitiligo

### 4.1 Changes in the number of peripheral blood treg

The frequency of Treg in the peripheral blood of patients with vitiligo remains controversial; however, most studies have reported a significant reduction. A meta-analysis incorporating multiple studies clearly demonstrated that the frequency of peripheral blood Treg was significantly decreased in patients with vitiligo (SMD = −1.26, *p* = 0.01) (12). Another large-scale meta-analysis further confirmed this finding and indicated that the reduction in Treg frequency was more pronounced in patients with active disease than in those with stable disease (14). Multiple independent studies have also supported the conclusion that the number of peripheral blood Treg is reduced (15). However, a minority of studies have reported no significant difference in peripheral blood Treg frequency compared with healthy controls (16), and some have even suggested an increasing trend, which may be related to heterogeneity in study populations, disease stage, and detection methods (17). Using higher-resolution single-cell sequencing technology, Xiao et al. observed in the peripheral blood of patients with progressive vitiligo that although the overall frequency of Treg may vary across studies, the number of cells within the STAM^+^ Treg subset was significantly increased at the subset level. This finding suggests that previous controversies regarding Treg cell numbers may, at least in part, stem from the inability to distinguish functionally distinct Treg subsets, and that expansion of specific subsets may reflect a compensatory or functionally dysregulated response during disease progression (4).

### 4.2 Functional and molecular phenotypic abnormalities of peripheral blood treg

Although changes in cell number remain inconsistent, functional impairment of peripheral blood Treg in patients with vitiligo is a well-established consensus. Their capacity to suppress the proliferation and activation of effector T cells (Teff), particularly CD8^+^ T cells, is markedly diminished (14, 18), and this functional defect is closely associated with aberrant expression of key Treg transcription factors and effector molecules.

First, the core transcription factor FOXP3 of Treg is significantly downregulated at both the mRNA and protein levels (12, 14, 17–19). Second, the levels of immunosuppressive cytokines secreted by Treg, such as TGF-β and IL-10, are significantly decreased in the serum (14, 17, 18), and intracellular IL-10 expression in Treg is also reduced (18). In addition, the soluble form and mRNA expression of immune checkpoint molecules such as CTLA-4 are decreased (17, 18). Functional studies have further shown that the proliferative capacity of Treg derived from patients with vitiligo is itself impaired (18).

From the perspective of cellular subsets, the internal composition of the peripheral blood Treg compartment is also altered. One multi-omics study identified a dysfunctional memory Treg (mTreg) subset characterized by expression of the skin-homing receptor cutaneous lymphocyte-associated antigen (CLA) and programmed cell death protein 1 (PD-1), together with impaired secretion of IL-4 and IL-13; this defect was particularly prominent in patients with active disease (20). Another study found that the proportion of Th1-like Treg in the peripheral blood of patients was significantly increased. This subset was characterized by expression of T-bet and IFN-γ, but low expression of CTLA-4 and IL-10, exhibited enhanced migratory responsiveness to the chemokines CXCL10 and CXCL16, and its expansion was considered an important contributor to the overall decline in Treg suppressive function (16). Beyond Th1-like Treg and dysfunctional mTreg, single-cell transcriptomic analyses have further revealed intrinsic molecular defects in Treg. Xiao et al. found that although the STAM^+^ Treg subset was increased in the peripheral blood of patients, these cells highly expressed STAT1 and were enriched in tumor necrosis factor (TNF)-related signaling pathways. At the transcriptomic level, this finding confirms a tendency of Treg to polarize toward a proinflammatory Th1-like phenotype, suggesting that these cells themselves may have acquir.

## 5 Studies on the infiltration and function of treg in progressive lesional tissue (skin)

### 5.1 Quantity and distribution of treg in skin tissue

Compared with the relatively heterogeneous findings in peripheral blood, the reduction in Treg cell number within skin tissue is more consistently observed, although differences remain in their regional distribution. Most studies have shown that, compared with healthy skin, the number of Treg is significantly decreased in both lesional and perilesional skin of patients with vitiligo (12, 21, 22). In animal models, the number of cutaneous Treg is negatively correlated with the degree of depigmentation (13). Large-scale meta-analyses have likewise confirmed downregulation of FOXP3 expression in the skin, particularly within lesional areas (14). Nevertheless, a small number of studies have reported inconsistent findings, such as an increased number of Treg in perilesional skin (12).

At the subset level, studies have demonstrated that both the frequency and absolute counts of tissue-resident memory Treg (TRM Treg) and antigen-specific Treg are significantly reduced in the skin (21). At the same time, the numbers of CD4^+^ and CD8^+^ tissue-resident memory T cells (TRM) are markedly increased. This reciprocal alteration—namely, a reduction in regulatory TRM cells (TRM Treg) accompanied by an expansion of effector TRM cells—directly results in an imbalance in the Teff/Treg ratio (21). In addition, immunofluorescence studies have confirmed the presence of Foxp3^+^T-bet^+^ Th1-like Treg in vitiligo lesional skin, and their abundance exceeds that of conventional Treg (16).

### 5.2 Functional status and homing of treg in skin tissue

Cutaneous Treg are characterized not only by insufficient numbers but also by profound functional impairment. Tissue-resident memory Treg (TRM Treg) and antigen-specific Treg isolated from patients’ skin exhibit a reduced capacity to secrete IL-10 and TGF-β and are therefore unable to effectively suppress the activation and proliferation of effector TRM cells (21). The local cutaneous microenvironment is skewed toward a proinflammatory state, as evidenced by upregulation of inflammatory mediators such as IL-15 and IL-17A and downregulation of anti-inflammatory factors such as TGF-β and IL-10, which is unfavorable for the maintenance and function of Treg (21). In addition, the expression of multiple Treg-associated immunosuppressive molecules, such as CTLA-4 and HO-1, as well as chemokine receptors including CCR5 and CCR6, is decreased in lesional skin (22).

The homing of Treg to inflamed skin is a prerequisite for their protective function. Studies have shown that the chemokine receptor CCR6 is critical for Treg migration to vitiligo lesions, and Treg lacking CCR6 are unable to effectively suppress disease progression (13). Although chemokine receptor expression is generally downregulated in Treg from patients, the Th1-like Treg subset highly expresses CXCR3 and CXCR6 and may therefore be more readily recruited into the skin (16). At the same time, reduced expression of the key Treg-attracting chemokine CCL22 in the local skin microenvironment may further restrict the recruitment of functional Treg (17). Interestingly, one study found that the abundance of Treg in perilesional skin was positively correlated with the number of CD8^+^ T cells, suggesting that Treg may indeed be recruited to sites of inflammation, but that their number and/or function is insufficient to fully suppress the ongoing autoimmune response (12).

### 5.3 Summary: from peripheral blood to skin—a systemic perspective on treg dysregulation

Taken together, the abnormalities of Treg in vitiligo are characterized by the coexistence of “numerical reduction” and “functional deficiency, “ and this dysregulation differs significantly between peripheral blood and lesional skin, suggesting a potential anatomical hierarchy (e.g., blood → non-lesional skin → lesional skin) that remains to be validated. Future studies incorporating non-lesional skin samples are needed to determine whether a true gradient exists or whether the observed differences simply reflect dichotomous systemic versus local dysfunction. In the peripheral blood, the overall frequency of Treg tends to decline, while functionally they demonstrate impaired suppressive capacity, reduced expression of key inhibitory molecules, and the emergence of dysfunctional subsets, such as Th1-like Treg and functionally exhausted memory Treg (mTreg) cells. In lesional skin, Treg—particularly those with tissue-resident and antigen-specific properties—are markedly reduced in number and exhibit impaired suppressive function, while being further compromised by the proinflammatory microenvironment. In addition, the homing capacity of Treg to the skin is impaired due to abnormalities in chemokine receptor/ligand axes. Collectively, these defects result in insufficient suppression of autoreactive CD8^+^ T cells, ultimately leading to melanocyte destruction. These abnormalities are often more pronounced in patients with active disease (14, 15, 18, 20), suggesting that Treg dysfunction is closely associated with disease progression. However, merely describing the numerical and functional defects of Treg themselves is still insufficient to fully explain why melanocytes become the specific targets of immune attack and why local immune tolerance fails to be re-established.

While the preceding sections have established that Treg in vitiligo exhibit profound numerical and functional defects in both peripheral blood and lesional skin, it remains unclear why these defects lead specifically to melanocyte destruction rather than a generalized loss of immune tolerance. Emerging evidence suggests that the breakdown of immune privilege in the melanocyte niche, together with shared signaling pathways that regulate both Treg function and melanocyte survival, may provide the missing link between Treg dysregulation and targeted melanocyte loss. The following section explores this Treg–melanocyte network and its implications for disease pathogenesis.

## 6 Treg–melanocyte interactions: a critical node in the breakdown of immune tolerance

**Figure 1:**
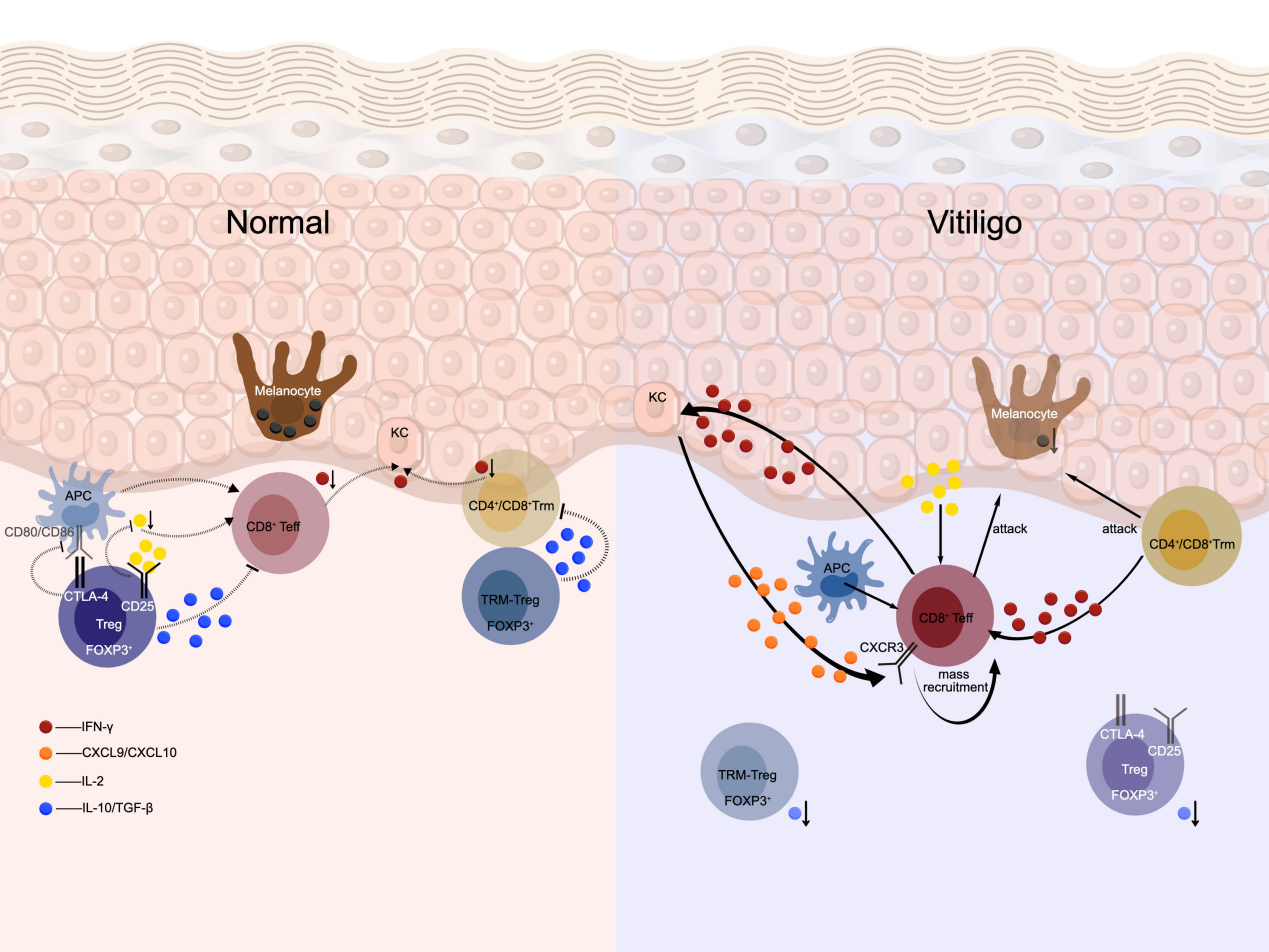
Comparison of the Treg-melanocyte immune network between normal skin and vitiligo skin.

In the pathogenesis of vitiligo, the systemic collapse of the dynamic and multilayered protective interaction network between Treg and melanocytes is considered one of the core mechanisms underlying the selective destruction of melanocytes. This disequilibrium does not arise from a single defect; rather, it represents a cascading process that progresses from systemic immune dysregulation to the failure of the local microenvironment and, ultimately, to the breakdown of target-cell homeostasis (**Figure 1**).

First, at the systemic and circulatory levels, fundamental defects in both the number and function of Treg already exist, thereby laying the groundwork for failure of this interactive network. Most studies have shown that the frequency of Treg in the peripheral blood of patients is significantly reduced, particularly during the active stage of disease (12). More importantly, Treg exhibit clear functional impairment, including a reduced capacity to suppress the proliferation and activation of CD8^+^ T cells, as well as downregulation of the key transcription factor FOXP3 and effector molecules such as TGF-β, IL-10, and CTLA-4 (12). This systemic functional impairment of Treg has been further substantiated at the single-cell transcriptomic level. The study by Xiao et al. not only identified the numerically altered STAM^+^ Treg subset, but more importantly, functional enrichment analysis revealed that this subset was involved in osteoclast differentiation, Epstein–Barr virus infection, TNF signaling, and IL-17 signaling pathways. Dysregulation of these pathways, particularly enrichment of TNF signaling, is consistent with the concept that Treg undergo polarization toward a Th1-like phenotype, indicating that not only are Treg intrinsically dysfunctional, but the broader immunoregulatory networks in which they participate have also become systemically disordered, thereby establishing the systemic basis for subsequent collapse of the local “protective umbrella” within the tissue microenvironment (4). Multi-omics analyses further confirmed the presence of a “dysfunctional Treg/mTreg response” in patients, in which skin-homing (CLA^+^) memory Treg (mTreg) cells display a persistently activated state characterized by high PD-1 expression, yet exhibit impaired secretion of the suppressive cytokines IL-4 and IL-13, consistent with an exhausted or dysfunctional phenotype (14). This suggests that even when Treg are recruited to the skin, they may already be functionally compromised.

Second, within the local cutaneous microenvironment, dysfunctional Treg fail to establish a “protective umbrella” against immune attack and may instead contribute to the amplification of inflammation. Compared with the relatively heterogeneous findings in peripheral blood, the reduction in Treg cell number within lesional skin is more consistently observed (12). More importantly, local functional disequilibrium is evident: Treg subsets with skin-resident and antigen-specific properties are reduced, whereas proinflammatory tissue-resident memory T cells (TRM) expand, resulting in a reversal of the effector/regulatory balance (20). These local Treg exhibit severely impaired suppressive function and are unable to effectively secrete IL-10 and TGF-β to neutralize inflammation (20). Meanwhile, the local microenvironment is characterized by a highly inflammatory state, with upregulation of IFN-γ, TNF-α, and IL-15. These cytokines not only directly attack melanocytes, but may also “reprogram” Treg toward a dysfunctional Th1-like phenotype expressing T-bet and IFN-γ; such dysfunctional Th1-like Treg subsets have indeed been identified in lesional skin of patients (12). In addition, interactions between antigen-presenting cells such as monocytes and effector T cells are amplified through co-stimulatory pathways including OX40/OX40L, while Treg fail to adequately restrain the activity of these auxiliary pathogenic cells (14). Consequently, the local microenvironment shifts from a “teloregion niche” enriched in TGF-β and IL-10 to a hostile inflammatory battlefield dominated by IFN-γ and TNF-α, leaving melanocytes fully exposed to immune attack.

Furthermore, melanocytes themselves, as the other principal component of this interaction, may lose their capacity to actively maintain immune homeostasis while simultaneously activating intrinsic death programs. Evidence suggests that melanocytes are not entirely passive targets. For example, in melanoma, stressed melanocytes can actively modulate local immunity through expression of molecules such as PD-L1. In vitiligo, however, this active “immunomodulatory” or “distress-signaling” mechanism may already be defective. More importantly, melanocytes under stress appear to initiate irreversible cell death pathways. Recent studies have shown that the NLRP3 inflammasome in melanocytes from patients is excessively activated because of impaired autophagic degradation, which is associated with downregulation of β-TrCP1 and reduced K27-linked ubiquitination. This leads to pyroptotic cell death and the release of large amounts of proinflammatory mediators such as IL-1β, which not only directly kill melanocytes but also further exacerbate the local microenvironment, thereby establishing a vicious cycle (20).

Finally, the “bridge” connecting Treg and melanocytes becomes disrupted, preventing effective crosstalk between the two. On the one hand, homing of Treg to the skin is impaired, and the expression of Treg-attracting molecules such as CCL22 is reduced in the local skin environment (12). On the other hand, shared signaling pathways that are crucial for the survival and function of both cell types, such as the TGF-β and Wnt pathways, may undergo coordinated suppression (23). TGF-β is not only a functional cytokine of Treg, but also a key growth factor for melanocytes; similarly, the Wnt pathway is involved in both Treg stability and melanocyte regeneration and differentiation. Suppression of these pathways within the inflammatory microenvironment prevents the effective transmission of “protective signals” between immune cells and target cells, ultimately compromising both the protective role of Treg and the survival requirements of melanocytes.

In summary, the disequilibrium of Treg–melanocyte interactions in vitiligo represents a multilayered and progressively amplified pathological process. It originates from systemic Treg dysfunction, manifests as failure to establish a locally immunosuppressive microenvironment together with the formation of a proinflammatory milieu, is aggravated by the loss of melanocyte-intrinsic immunoregulatory capacity and activation of intrinsic death programs, and ultimately becomes irreparable because of homing defects and dysregulation of critical shared signaling pathways. The comprehensive collapse of this interaction network ultimately leaves melanocytes isolated and unprotected in the face of autoimmune attack, resulting in depigmentation.

## 7 Treg as therapeutic targets: clinical and experimental advances

### 7.1 Therapeutic strategies to promote treg

Based on the immunopathological framework of vitiligo, which is characterized by excessive activation of effector T cells and insufficient immune regulation, multiple recent reviews have proposed that enhancing the number of Treg or restoring their suppressive function may represent a potential therapeutic strategy for correcting immune disequilibrium in this disease. Among the currently available lines of evidence, the immunomodulatory effects of phototherapy, particularly narrowband ultraviolet B (NB-UVB), have been documented in multiple studies. NB-UVB phototherapy has been shown to significantly increase FOXP3 expression in vitiligo skin, suggesting that it may promote restoration of the Th17/Treg balance and thereby improve local immune tolerance (24). Further clinical immunological analyses have demonstrated that NB-UVB significantly reduces the frequencies of peripheral CD4^+^ and CD8^+^ central memory T cells as well as the levels of the proinflammatory chemokines CXCL9 and CXCL10, indicating that its overall immunosuppressive effects may indirectly create a more permissive environment for local Treg-mediated regulation (25).

JAK inhibitors are considered capable of creating conditions favorable for Treg functional recovery by attenuating the inflammatory milieu. The critical role of the IFN-γ–CXCL9/CXCL10–CXCR3 axis in sustaining cutaneous infiltration of CD8^+^ T cells and amplifying inflammation in vitiligo has been systematically summarized (11). On the basis of this mechanism, JAK inhibitors have been shown to markedly reduce the activation and skin recruitment of pathogenic CD8^+^ T cells by blocking IFN-γ–JAK/STAT signaling, thereby alleviating local inflammatory pressure (26). Although these agents do not directly target Treg cells, it has been proposed that amelioration of the inflammatory microenvironment may provide more favorable conditions for regulatory immune cells to regain local function (26). Single-cell transcriptomic studies have provided additional potential biological support for the application of JAK inhibitors. Xiao et al. found that the expanded STAM^+^ Treg subset in the peripheral blood of patients with vitiligo highly expressed STAT1 and was enriched in the JAK–STAT signaling pathway. STAM itself, as a JAK-associated signaling molecule, may participate in regulating the activity of this pathway. Therefore, JAK inhibitors may not only suppress the pathogenic functions of effector T cells but also potentially correct the Th1-like polarization state of Treg by interfering with intracellular JAK–STAT signaling, thereby “repairing” Treg function. This provides a deeper mechanistic interpretation of the immunomodulatory effects of JAK inhibitors (4).

In addition to the above strategies that indirectly improve the immune microenvironment, recent experimental and early clinical studies directly targeting Treg have also opened new therapeutic possibilities for vitiligo. Multiple clinical and mechanistic studies have demonstrated that low-dose IL-2 (LD-IL-2) therapy can selectively expand FOXP3^+^ regulatory T cells and enhance their suppressive function. In autoimmune settings such as chronic graft-versus-host disease, LD-IL-2 has been shown to significantly increase circulating FOXP3^+^ Treg cell numbers and restore immune tolerance, thereby providing a theoretical rationale for extending this strategy to other immune-mediated disorders, potentially including autoimmune skin diseases, by extrapolation from findings in other autoimmune conditions (27). Treg not only play a central role in maintaining immune tolerance in vitiligo, but also represent an important potential target for future immunotherapeutic intervention.

### 7.2 Treg as biomarkers of therapeutic response and prognosis

From the perspective of clinical study design, the key prerequisite for using Treg as predictors or monitors of therapeutic efficacy is to demonstrate that Treg-related indicators can dynamically change during treatment and that such changes show reproducible associations with repigmentation and/or disease control. A prospective exploratory study was conducted in patients with non-segmental vitiligo, in which lesional skin sampling and peripheral blood immune/proteomic monitoring were performed simultaneously during the early phase of treatment (28). The study suggested that, following therapy, CD8^+^ T cells and certain TRM-related cell populations in the skin decreased, while dynamic changes were also observed in the Tr1 subset and IL-10-secreting Tr1 cells in the peripheral blood; some of these immunological alterations were associated with subsequent repigmentation. This study highlights that “local cutaneous immune changes” during treatment are not always concordant with “peripheral blood changes.” Therefore, if Treg-related indices are to be used as biomarkers, both the “sampling site” and the “endpoint definition” must be clearly specified; otherwise, phenotypic mismatch is likely to occur.

Furthermore, as emerging therapies such as JAK inhibitors enter the stage of clinical investigation, an increasing number of trials have begun to incorporate immune biomarker endpoints to explain “who is likely to repigment” and “who is prone to relapse.” Accordingly, more convincing future evidence should come from longitudinal cohorts established under standardized treatment protocols, in which both the number and function of Treg—or related suppressive molecules, such as IL-10—are measured simultaneously and modeled in relation to repigmentation rate, duration of response maintenance, and risk of relapse after treatment discontinuation. The future development of Treg-related biomarkers should not be restricted to total Treg counts or FOXP3 expression alone, but should instead focus on functionally defined subsets with greater disease specificity. The STAM^+^ Treg subset identified by Xiao et al. using scRNA-seq, or the ratio of FCGR3A^+^ CD8^+^ T cells to STAM^+^ Treg, may represent novel peripheral blood biomarkers reflecting disease activity and therapeutic responsiveness. Monitoring the dynamic changes of these specific subsets before and after treatment may enable more precise prediction of repigmentation outcomes and relapse risk (4). This strategy will determine whether Treg can be advanced from a “mechanistic clue” to a clinically applicable biomarker variable.
